# Analysis of the Attitudes towards Sexuality in People with Intellectual Disabilities: A Cross-Sectional Study

**DOI:** 10.3390/nursrep13040134

**Published:** 2023-11-11

**Authors:** José Carlos López-García, Azucena González-Sanz, Elena Sutil-Rodríguez, Carlos Saus-Ortega, Regina Ruiz de Viñaspre-Hernádez, Raúl Juárez-Vela, Vicente Gea-Caballero, Juan Luis Sánchez-González, Clara Isabel Tejada-Garrido, Ana Cobos-Rincón, José María Criado-Gutiérrez, Consuelo Sancho-Sanchez

**Affiliations:** 1 Doctoral Program in Health, Disability, Dependence, and Welfare, University of Salamanca, 37008 Salamanca, Spain; josecarlosdue@usal.es; 2 Adscript Center of Zamora, School of Nursing, University of Salamanca, 37008 Zamora, Spain; agonzalezsa@usal.es (A.G.-S.); esutil@usal.es (E.S.-R.); 3 Adscript Center of La FE, School of Nursing, University of Valencia, 46003 Valencia, Spain; carles.saus-ortega@uv.es; 4 Faculty of Health Sciences, University of La Rioja, 26006 Logroño, Spain; clara-isabel.tejada@unirioja.es (C.I.T.-G.); ana.cobos@unirioja.es (A.C.-R.); 5 Faculty of Health Sciences, International University of Valencia, 46003 Valencia, Spain; vagea@universidadviu.com; 6 Faculty of Nursing and Physiotherapy, University of Salamanca, 37008 Salamanca, Spain; juanluissanchez@usal.es; 7 Faculty of Medicine, University of Salamanca, 37008 Salamanca, Spain; jmcriado@usal.es (J.M.C.-G.); sanchoc@usal.es (C.S.-S.)

**Keywords:** intellectual disability, sexuality, attitudes

## Abstract

The barriers faced by people with intellectual disabilities are many. One of the areas in which many problems have been identified is the sexual domain. This descriptive study aims to analyze the attitudes of the family environment, professional carers, and the general population toward their sexuality. A cross-sectional descriptive study was carried out between 2022 and 2023, using convenience sampling among family members and carers from different centers working with people with intellectual disabilities in Spain, and among the general population not related to people with intellectual disabilities. A total of 583 responses were received and significant differences were found for all variables, with the variables related to family or work proximity being those that provided the most significant and relevant results. It was observed that the male sex has a more paternalistic attitude and that in rural areas there is a more permissive attitude towards the sexuality of people with intellectual disabilities. People who work with people with disabilities have more positive attitudes towards this group, while direct relatives have more paternalistic attitudes. Nursing care in the community and specialized centers should be based on an adequate therapeutic relationship and personalized care.

## 1. Introduction

Intellectual disability (ID) is a concept that has evolved over the last few decades. These changes have affected the way the condition itself is described, to avoid linguistic degradation, but also its defining characteristics, which include people with below-average IQ, but with the addition of adaptive limitations to the environment, and all this limited to a diagnosis before the age of 22 [1]. Limitations related to social skills and adaptation to family, work, and social environments are a fundamental pillar of diagnosis [1]. The types and causes of ID are diverse and it is necessary to use psychometric tools to make a correct diagnosis and identify the main needs for its management from a multidisciplinary approach [2].

The barriers faced by people with ID are numerous, ranging from limitations inherent in their diagnosis to social or work limitations [3,4]. One of the fields in which numerous problems have been demonstrated in people with ID has been the sexual domain. Sexuality is inherent in human beings from the moment of conception. It affects all developmental domains and every social facet of our everyday life [5]. The way of living and manifesting sexuality is multiple. It is influenced by culture, education, and multiple personal and social aspects [6].

Sexuality is often associated with genitality [7]. A comprehensive view of sexuality considers several dimensions of sexuality, such as the biological, psychological, and social domains [8]. This broad vision is reflected in the WHO definition of sexuality [9], which refers to the multiple ways of feeling and expressing it through thoughts, fantasies, desires, beliefs, attitudes, values, behaviors, practices, roles, and relationships. Despite the importance of sexuality, rights in this area are effected in all cultures and by any physical or psychological change in the individual. Acute and chronic illnesses, the need for hospitalization and surgery, the use of multiple medications [10], and mental health problems of all kinds, especially chronic ones [11], hurt sexuality. Globally, there are still problems affecting sexuality that are difficult to address due to their social, cultural, and economic roots, such as trafficking in women [12], clitoral cutting [13], gender-based violence, sexual abuse, and pornography and the inclusion of new issues such as sexting [14], which limit freedom in the area of sexuality.

Many specific problems have been described concerning the experience of sexuality in the population with ID, such as difficulties in education, higher rates of sexual abuse, and changes in affectivity or perception, among others [15]. Many attempts have been made to identify the causes of these problems, and the solution has focused on improving the sexual education of people with ID [16]. However, an increasing number of studies have investigated attitudes towards and around people with ID as a source of problems in sexual matters for people with ID [17,18,19,20]. To analyze this situation, several surveys have been developed to determine the influence of these attitudes.

The role of nursing with the ID collective is little addressed. There is little research that discusses the roles of nursing in the team of people in contact with this collective [21,22].

The present study aims to find and apply a validated scale and to analyze the attitudes of different population groups according to their proximity to people with ID. We will consider assessing the influence of being a family member of a person with ID, the influence of being a caregiver of a person with ID, or having no relationship with a person with ID. The influence of sociodemographic factors will also be considered to determine the influence on attitudes toward the sexuality of people with ID.

## 2. Material and Methods

A cross-sectional, descriptive, and analytical study was carried out.

### 2.1. Setting and Sample

A multicenter study was conducted in Spain and there were no exclusion criteria. The study was conducted between October 2022 and January 2023.

Professionals from centers working with people with intellectual disabilities, family members of people with intellectual disabilities, and the general population were invited to participate in the study. To select the sample, cluster sampling was carried out among the different associations working with groups of people with ID, asking them to disseminate the survey among their employees and family members. The sample of professionals and relatives of people with ID was recruited in different professional centers, preferably in Castilla-León, but also in other Autonomous Communities of Spain, which were contacted and informed about the study. To obtain the sample of the general population, non-probabilistic snowball sampling was performed, using different digital media to complete the questionnaire.

### 2.2. Tool

A literature search was conducted to find a suitable questionnaire on the attitudes of the population towards the sexuality of people with ID.

The search was conducted in the Web of Science and PubMed databases in September 2022. The search was limited to articles published in the last 20 years in Spanish, English, and Portuguese. The terms “intellectual disability”, “scale”, and “attitude” were used in the search, and the Boolean operators “AND” and “OR” were used. The initial reference search was performed by the first author of the manuscript and 526 articles were found. The selection and search for scales was carried out by the second and third authors of the manuscript, after reading the titles and abstracts. The scales should meet certain criteria such as “as up-to-date as possible, with a limited number of questions, easy to complete and disseminate, and covering all dimensions of sexuality, not only sexual relations”. The literature search provided three scales, namely the Perception of Sexuality Scale (POS) [23], the questionnaire on attitudes towards sexuality in people with intellectual disabilities (ASQ-ID) [24], and the questionnaire Attitudes Towards Sexuality of Individuals with Intellectual Disability (ASEXID) [25].

To select the most appropriate questionnaire, five experts in ID were asked to analyze the questionnaires and give a score from 0 to 10 according to five criteria of the scale: appropriateness of the number of items, adaptability to the study population, breadth of topics addressed by the questionnaire about sexuality, comprehension of the questions, and speed of response. After analyzing the different questionnaires, the ASEXID questionnaire [25] (Assessment of attitudes towards the sexuality of people with intellectual disabilities) was selected. This questionnaire assesses attitudes towards sexuality in people with ID through 18 items with a Likert-type response format with five levels of frequency (from strongly disagree to strongly agree). This questionnaire has been validated in Spanish among professionals and family members of people with ID and the general population.

The 18 items are grouped into three factors: normalizing attitudes, expressing the similarity between people with and without ID; denying attitudes, expressing a lower sexual desire in people with ID; and paternalistic attitudes, expressing a perceived lack of impulse control in people with ID. This questionnaire is designed to be completed by people with family members with ID, caregivers of people with ID, and the general population. In addition to the ASEXID questionnaire, five short questions on some sociodemographic characteristics are requested for further analysis, which are:

1. Whether they have a direct family member with ID.
2. Whether they are a caregiver of a person with ID.
3. Their age, which is grouped into several intervals (0–20, 20–40, 40–60, and over 60).
4. Their sexual identity: male or female.
5. Whether they live in rural or urban areas.

### 2.3. Ethical Considerations

This study was approved by the Bioethics Committee of the University of La Rioja, which issued a favorable report with verification URL: https://sede.unirioja.es/csv/code/p2Cc6Wk1S6UT6lszlr3Gg9ukhS3Ey7ha (accessed on 5 July 2023).

Furthermore, the study was conducted following the principles of the Declaration of Helsinki and good clinical practice. Reporting was performed following the guidelines for strengthening the reporting of observational studies in epidemiology (STROBE) [26].

### 2.4. Procedure

After being fully informed about the purpose of the study and giving their informed consent, participants were asked to complete an anonymous questionnaire through the Google Forms ^®^ platform. Contact with family members and workers was made through the directors of the centers working with people with ID, who facilitated access to the questionnaire online. The distribution of the questionnaire to the general population was carried out through the use of social networks.

### 2.5. Statistical Analysis

We established a 95% confidence interval by entering all the variables in the SPSS v.27 program and analyzing the characteristics of the responses given, as well as the relationship between the different variables. The Kolmogorov–Smirnov test [27] and visual inspection of the histograms were performed to evaluate the distribution of the data (*p* values < 0.05 were considered non-normal, a result obtained for some of our variables).

With the results of the Kolmogorov–Smirnov test and taking into account the characteristics of the variables to be analyzed, the Mann–Whitney U test [28] was used for the independent variables: the presence of a direct relative, the gender of the respondent, the caregiver of a relative with ID, or the place of residence. For the age analysis, Spearman’s Rho [29] and Kendall’s Tau c [30] were used, which were determined to be the most appropriate tests, since both variables are ordinal.

## 3. Results

The total number of responses was 583. Most of the people who accessed the survey agreed to answer it (99.1%). Despite the difficulty in finding direct relatives or caregivers of persons with ID, 7.4% and 13.9% of the responses were obtained from these groups of participants, respectively. The number of participants who had no professional or personal relationship with a person with ID was 459 (78.7%). The age of respondents varied, but the vast majority (85.4%) were between 20 and 60 years old. Most respondents lived in urban areas (81.4%). The number of women was higher at 67.7% compared to 27.4% who were men. The sociodemographic characteristics of the participants are shown in Table 1.

In general, the highest percentage of responses to the questionnaire were grouped in one of the extremes of the Likert scale (“Strongly disagree” or “Strongly agree”). If we add to the majority response the closest response on the Likert scale, the sum of both responses exceeds 75% of the total responses in most questions. However, there are several exceptions:

1. In question 7 questioning whether people with ID can control their sexual urges, the middle three responses reached 87.6%.
2. In question 11 on whether they agreed that people with ID can have sex without penetration, 71.6% of respondents disagreed (“Strongly disagree” or “Somewhat disagree”).
3. In question 13 on whether people with ID need a guardian to decide their sexuality, the responses were almost equally distributed between “Strongly disagree”, “Somewhat disagree”, and “Neither agree nor disagree” answers.
4. Questions 15, 16, and 18 have the largest differences between their responses. The biggest difference between one response and another is seen in question 15 on whether people with ID see the danger of sexual abuse. In question 16 on whether they are okay with people with ID viewing pornography, the majority take a neutral stance, although they are more in favor of yes. The question with the greatest degree of dispersion is question 18 (“A woman with ID should be prevented from getting pregnant by using contraception”), with 1/3 of people in the neutral zone, 1/3 choosing “Agree” or “Strongly agree”, and 1/3 selecting “Somewhat disagree” or “Strongly disagree” (Table 2).

If we analyze the relationship between the responses to the ASEXID scale and demographic characteristics, we find that highly significant differences are found in the variables “Direct family of people with ID”, “Professional caregiver of people with ID”, and in the different “Age groups”. The analysis of the variables is shown in Table 3.

With the variable “gender”, a high significant difference is obtained in question 1 (People with ID have less interest in sexuality than people without ID) with a *p* of 0.024 and in question 3 (Talking about sex with people with ID is to encourage them to practice it) with a *p* of 0.003. In both questions, it is men who consider that people with ID have less sexual desire.

The variable “age” had the highest number of significant differences in 11 of the 18 questions, with a high degree of significance in 9 of them, with values lower than *p* < 0.01. To evaluate the association between these ordinal variables, Spearman’s Rho and Kendall’s Tau c were used. In both tests, the results were similar, but when interpreting Spearman’s correlation coefficient, it can be observed that it is very low or low in all cases. When analyzing the age groups, in the questions related to affective aspects, friendship, or courtship, it is the older groups that have a more permissive attitude towards IDs, while in the questions related to explicit sexual relations or parenting, the older groups have a more paternalistic attitude (which is defined as the tendency to apply the father’s forms of authority and protection in the traditional family to other forms of social relations: political, labor, etc.).

In the variable “Place of residence”, significant differences are obtained in questions 13 (People with ID need a guardian) and 15 (People with ID perceive sexual abuse) with degrees of significance of 0.036 and 0.009, respectively, and in which a more “paternalistic” attitude of people residing in urban versus rural areas is observed since they consider a higher percentage of people in urban areas do believe that they should have a guardian and that they do not perceive sexual abuse.

The variable “Professional caregiver of people with ID” shows a significant number of significant results in questions that include the three factors analyzed in the ASEXID scale (normalizing attitude, negative attitude, and paternalistic attitude), and all of them reflect a more favorable attitude of professional caregivers towards the sexuality of people with ID than the general population or family groups, except for question 15 where they think that people with ID do not perceive sexual abuse.

The variable “Direct relative of a person with ID” is highly significant in pre-questions 9, 10, and 12, which evaluate the factor “Normalizing attitude”. In all of them, it is worse than in the group of caregivers and the general population. Question 15 increased with the group of caregivers and both thought that people with ID do not perceive the risk of sexual abuse in comparison with the general population.

## 4. Discussion

We present a novel study on the topic. Significant differences were found in all the variables analyzed: proximity to people with ID is a determining factor and what most determines the level of protection and attitudes towards this group is kinship.

The first analysis suggested by this study is the percentage of female respondents compared to male respondents. The sample search did not focus on predominantly female groups; however, the 3:1 response rate suggests that we should analyze whether this is a recurring circumstance in many surveys and why, or whether it is specific to this topic.

As with gender, there is a significant difference between caregivers and family members of people with ID. The survey was distributed in a significant number of organizations; if we take into account the number of workers caring for people with ID, which will always be lower than the number of people with ID, and the number of family members, which will always be much higher, we might think that the responses of family members would exceed those of caregivers. However, twice as many caregivers than family members responded.

The variable “gender” shows only two responses with significant differences in both cases: it is the male gender that shows a more conservative response to sexuality in ID, a result that coincides with other studies [18,19,20,29].

A study conducted in Australia in 2009 evaluated the same population groups (family members, caregivers, and the general population) and found that fathers were more conservative than workers about sexuality, and both were more conservative than the rest of society concerning parenting. Age also represented a difference in attitudes, with people over 60 also being more conservative. They relate this to the fact that in many cases the parents are older than 60 years and it is not specified whether this applies to all questions or only to some [18]. The same scale was used in a 2015 study in England to measure attitudes toward the sexuality of people with ID, but in this case, different cultural groups were compared, determining that people of Asian origin are less considerate and have greater social control vis-à-vis the sexual rights of people with ID [19].

Other scales, such as the ASQ-GD (for the general population) and the ASQ-ID (for people with ID) used in Australia in 2010, assessed the attitudes of leisure workers towards sexuality in people with ID. The results showed a generally positive attitude, although they noted that men had less self-control, thought that women with ID should have less sexual freedom, and were very cautious about their attitude toward parenting in this group [20]. Another study conducted in Australia in 2012 using the same scale suggests that sexuality training may benefit direct care workers with ID, especially older female workers [31]. A meta-analysis of articles using the ASQ-ID scale was conducted in 2022 [32].

The POS Sexuality Perception Scale in a 1996 study in Alaska showed that college students viewed the sexual behavior of people with ID as less acceptable than their own [33].

## 5. Nursing Clinical Implications

The role of the nurse in this group is little known. However, nurses care for this type of patient in the community setting for primary care and are present in most specialized centers working with people with ID. Two literature reviews [21,22] stand out in which the key role of the nurse in the multidisciplinary team that cares for these people is identified, focusing attention on establishing appropriate therapeutic communication and individualizing the care plan according to personal needs. Nursing is key to enabling people with ID to adapt to their environment holistically: facilitating the development of social skills, teaching new cognitive skills to enable information processing and problem-solving, and creating new tools to enhance learning in new situations of daily living.

## 6. Study Limitations

The main limitation found in the study is the lack of response from people who are part of the ID environment. About 100 organizations involved in the care of people with ID were contacted to disseminate the survey among workers and their families, but the number of responses was low to the number of organizations contacted, and this is even though the survey was completely anonymous, brief, and concrete. There are even organizations that have refused to disseminate the survey because it deals with a topic related to sexuality in ID on the part of the organizations or at the request of partners or family members. These data cannot be reflected in the study, as they have not been quantified or analyzed, but they suggest comprehensive analysis in the future to determine the importance of this refusal in this and other topics related to ID. In general, the samples in all the studies analyzed are not very large.

## 7. Conclusions

With the “gender” variable, a more paternalistic attitude can be observed in the male population compared to the female population.

Regarding the place of residence, there are no previous studies to compare and in this case, the data show a more paternalistic attitude in the urban population compared to the rural population.

The younger age groups have a more paternalistic attitude in matters of friendship or courtship; however, this attitude is reversed and it is the older population groups that have a more paternalistic attitude in matters of more explicit sex or in matters related to the upbringing of people with ID.

It can be affirmed that the group of caregivers is more concerned about this issue, responding to this survey in a higher percentage, and they present a more respectful attitude compared to the group of relatives, who have a more paternalistic attitude.

It is suggested that similar studies be carried out with a larger sample size to extrapolate the results and implement them in practice in the future to achieve a decrease in sexual complications in people with ID and improve their level of satisfaction in this area.

Most studies and actions focus on the people closest to the people with ID, but more actions should be carried out in the general population so as not to stigmatize the group. It would be interesting to assess whether these actions would have an impact on the attitudes of family members toward the sexuality of people with ID.

## Figures and Tables

**Table 1:** Sociodemographic characteristics of the participants.

		N = 583	
		N	%
Gender	Male	160	27.4%
	Female	395	67.7%
	No answer	28	4.8%
Age	0–20 years old	46	7.9%
	20–40 years old	261	44.8%
	40–60 years old	219	37.6%
	+60 years old	36	6.2%
	No answer	21	3.63%
Residence	Urban	459	78.7%
	Rural	105	18%
	No answer	19	3.3%
Professional carers of people with intellectual disabilities	Yes	81	13.9%
	No	494	84.7%
	No answer	8	1.3%
A direct relative of a person with ID	Yes No	43 532	7.4% 91.2%
	No answer	8	1.4%

**Table 2:** Analysis of ASEXID scale responses.

	Likert Scale Responses					
Questions	1 Strongly Disagree	2 Somewhat Disagree	3 Neither Agree nor Disagree	4 Agree	5 Strongly Agree	No Answer
People with ID have less interest in sexuality than people without ^2^	306 52.5%	146 25%	98 16.8%	22 3.8%	4 0.7%	7 1.2%
2. Sexual education should only be provided to people with ID when they demand it ^2^	436 74.8%	107 18.4%	15 2.6%	10 1.7%	7 1.2%	8 1.4%
3. Talking to people with ID about sex encourages them to practice it ^2^	425 72.9%	92 15.8%	48 8.2%	8 1.4%	3 0.5%	7 1.2%
4. Masturbation can harm people with ID ^2^	498 85.4%	43 7.4%	22 3.8%	7 1.2%	3 0.5%	10 1.7%
5. It seems good to me that people with ID masturbate ^1^	12 2.1%	5 0.9%	95 16.3%	174 29.8%	289 49.6%	8 1.4%
6. A person with ID can live their sexuality as anyone else ^1^	14 2.4%	59 10.1%	38 6.5%	204 35%	260 44.6%	8 1.4%
7. People with ID can control their sexual impulses ^3^	10 1.7%	139 23.8%	225 38.6%	147 25.2%	52 8.9%	10 1.7%
8. People with ID should have their privacy ^1^	1 0.2%	4 0.7%	8 1.4%	147 25.2%	413 70.8%	10 1.7%
9. People with ID can have a partner ^1^	1 0.2%	5 0.9%	15 2.6%	165 28.3%	387 66.4%	10 1.7%
10. It seems good to me that people with ID kiss or caress with another person ^3^	2 0.3%	4 0.7%	25 4.3%	149 25.6%	393 67.4%	10 1.7%
11. It seems good to me that people with ID have sex as long as there is no penetration ^3^	289 49.6%	128 22%	123 21.1%	22 3.8%	11 3.9%	10 1.7%
12. It seems good to me that people with ID have sexual intercourse even with penetration ^1^	6 1%	9 1.5%	73 12.5%	210 36%	276 47.3%	9 1.5%
13. People with ID need another adult guardian to decide about their sexuality ^3^	183 31.4%	170 29.2%	142 24.4%	65 11.1%	13 2.2%	10 1.4%
14. People with ID are always heterosexual ^2^	406 69.6%	90 15.4%	40 6.9%	3 0.5%	3 0.5%	10 1.7%
15. People with ID perceive the danger of sexual abuse ^3^	81 13.9%	198 34%	165 28.3%	96 16.5%	34 5.8%	9 1.5%
16. It is normal for people with ID to see pornography ^1^	41 7%	50 8.6%	231 39.6%	167 28.6%	84 14.4%	10 1.7%
17. People with ID can use condoms properly to prevent infections ^3^	4 0.7%	23 3.9%	67 11.5%	195 33.4%	284 48.7%	10 1.7%
18. We should prevent women with ID from becoming pregnant through the use of contraceptives ^3^	86 14.8%	83 14.2%	201 34.5%	132 22.6%	74 12.7%	7 1.2%

^1^ Normalizing attitude that expresses the similarity between people with and without ID. ^2^ Denying attitude that expresses a lower sexual desire in people with ID. ^3^ Paternalistic attitude that expresses a supposed lack of impulse control in people with ID.

**Table 3:** Analysis of socio-demographic variables and ASEXID questionnaire.

	Sex	Age	Place of Residence	Professional Carer	Immediate Family ID
	*p*				
Question					
People with ID have less interest in sexuality than people without ^2^	0.024 *	0.011 *	0.543	<0.001 *	0.531
2. Sexual education should only be provided to people with ID when they demand it ^2^	0.209	0.444	0.600	0.124	0.706
3. Talking to people with ID about sex encourages them to practice it ^2^	0.003 *	0.291	0.231	0.090	0.314
4. Masturbation can harm people with ID ^2^	0.335	0.354	0.334	0.115	0.305
5. It seems good to me that people with ID masturbate ^1^	0.577	<0.001 *	0.769	0.002 *	0.153
6. A person with ID can live their sexuality as anyone else ^1^	0.423	0.059	0.636	0.012 *	0.053
7. People with ID can control their sexual impulses ^3^	0.644	0.214	0.125	0.577	0.249
8. People with ID should have their privacy ^1^	0.456	<0.001 *	0.241	0.01 *	0.137
9. People with ID can have a partner ^1^	0.111	<0.001 *	0.445	0.022 *	0.017 *
10. It seems good to me that people with ID kiss or caress with another person ^3^	0.648	<0.001 *	1.000	0.26	0.002 *
11. It seems good to me that people with ID have sex as long as there is no penetration ^3^	0.070	0.177	0.192	0.458	0.635
12. It seems good to me that people with ID have sexual intercourse even with penetration ^1^	0.143	<0.001 *	0.616	0.047 *	0.007 *
13. People with ID need another adult guardian to decide about their sexuality ^3^	0.097	<0.001 *	0.036 *	0.076	0.505
14. People with ID are always heterosexual ^2^	0.70	0.036 *	0.744	0.02 *	0.947
15. People with ID perceive the danger of sexual abuse ^3^	0.118	<0.001 *	0.009 *	0.014 *	0.038 *
16. It is normal for people with ID to see pornography ^1^	0.687	<0.001 *	0.998	0.008 *	0.452
17. People with ID can use condoms properly to prevent infections ^3^	0.139	0.294	0.652	0.545	0.423
18. We should prevent women with ID from becoming pregnant through the use of contraceptives ^3^	0.460	<0.001 *	0.333	0.314	0.728

^1^ Normalizing attitude that expresses the similarity between people with and without ID. ^2^ Denying attitude that expresses a lower sexual desire in people with ID. ^3^ Paternalistic attitude that expresses a supposed lack of impulse control in people with ID. * *p* < 0.05 are considerate with statistical signitication U-Mann-Whitney and Rho Spearman test.

## Data Availability

Data will be available by contacting the first author.

## Public Involvement Statement

There has been no public involvement in any aspect of this research.

## Guidelines and Standards Statement

This manuscript was drafted against the STROBE guidelines for Observational Studies.
